# Immunoinformatics‐Based Design of a Multi‐Epitope Vaccine Targeting a Conserved Copper–Associated Protein in *Neisseria gonorrhoeae*

**DOI:** 10.1002/bab.70179

**Published:** 2026-05-08

**Authors:** Sinethemba H. Yakobi, Uchechukwu U. Nwodo

**Affiliations:** ^1^ Patho‐Biocatalysis Group (PBG), Department of Biochemistry and Biological Sciences University of Fort Hare Alice South Africa

**Keywords:** conserved antigen, copper homeostasis, epitope prediction, immunoinformatics, multi‐epitope vaccine, *Neisseria gonorrhoeae*

## Abstract

*Neisseria gonorrhoeae* poses an urgent public health challenge due to rapidly increasing antimicrobial resistance and the absence of an effective vaccine. Targeting conserved bacterial pathways involved in essential physiological processes may provide new opportunities for vaccine antigen discovery. In this study, we applied an immunoinformatics‐based pipeline to explore a conserved copper–associated protein as a potential antigenic source for multi‐epitope vaccine design. Analysis of 396 clinical genomes confirmed high assembly quality and identified a highly conserved hypothetical protein (AKOBGLPP_01618) through proteome‐wide screening for metal‐binding signatures. The candidate exhibited strong predicted antigenicity, high prevalence across isolates (99.5%), and no detectable homology with human proteins. In silico analyses suggested potential copper‐binding features based on predicted metal‐coordinating residues and conserved genomic context. Epitope mapping identified B‐cell epitope–rich regions that were refined into conserved, surface‐accessible peptides. Predicted cytotoxic and helper T‐cell epitopes were filtered for immunogenicity, safety, and strain conservancy prior to inclusion in a multi‐epitope construct containing a TLR4‐targeting adjuvant and processing‐optimized linkers. The resulting 155‐amino‐acid construct demonstrated favorable physicochemical characteristics, predicted solubility, and an estimated global population coverage of 74.84%. Docking analyses suggested a consistent interaction propensity with the TLR4/MD‐2 complex across multiple predicted conformations. Although these findings provide preliminary computational support for the proposed construct, experimental validation will be required to confirm antigen function, immunogenicity, and receptor engagement. Collectively, this study identifies a conserved copper–associated protein as a potential antigen source and presents a computational framework for exploring vaccine candidates against *N. gonorrhoeae*.

## 1 Introduction

*Neisseria gonorrhoeae*, the causative agent of gonorrhea, represents an escalating global public health challenge [1]. As a World Health Organization (WHO) priority pathogen, treatment is increasingly compromised by the rapid emergence of antimicrobial resistance (AMR), which threatens the effectiveness of multiple first‐line antibiotics. Beyond acute urogenital disease, untreated or recurrent infections can result in severe complications, including pelvic inflammatory disease, ectopic pregnancy, infertility, and increased susceptibility to HIV transmission [2]. Despite the substantial disease burden, no licensed vaccine currently exists, highlighting a critical gap in preventive strategies [3]. Traditional vaccine strategies against *N. gonorrhoeae* have faced significant hurdles, primarily due to the pathogen's extensive antigenic variation of surface structures like pili and opacity (Opa) proteins and phase variation mechanisms that facilitate immune evasion [4, 5]. This has shifted the paradigm in gonorrhea vaccinology towards targeting highly conserved, essential antigens involved in core physiological processes that are less susceptible to variation. One such promising avenue is the targeting of bacterial metal homeostasis machinery [6, 7]. Copper, while an essential cofactor for key enzymes like the nitrite reductase AniA and cytochrome c oxidase, is potently toxic in excess. Consequently, gonococci have evolved sophisticated systems for copper acquisition, trafficking, storage, and efflux to maintain optimal intracellular levels [8]. The copper storage protein (Csp) family in other bacteria sequesters excess cytosolic copper, mitigating toxicity and serving as a reservoir for cuproenzymes [8, 9]. In *N. gonorrhoeae*, while canonical copper exporters (CopA) and chaperones are characterized, a dedicated, conserved Csp remains elusive and represents a potential Achilles' heel—an essential, conserved component of a stress‐response pathway critical for survival in host environments, such as the copper‐rich phagosome [10]. Targeting such a protein could disrupt a fundamental homeostatic process, impairing bacterial fitness and virulence. Advances in reverse vaccinology and immunoinformatics have enabled genome‐wide identification of conserved vaccine targets. These approaches allow computational screening of pathogen proteomes to identify antigenic proteins and design multi‐epitope vaccines (MEVs) composed of selected B‐cell and T‐cell epitopes [11, 12]. By focusing immune responses on conserved and immunogenic regions while excluding non‐protective sequences, MEVs offer several advantages, including improved specificity, modular design, and suitability for rapid development [13]. This strategy is particularly suited for pathogens like *N. gonorrhoeae*, where defining conserved, protective antigens has been challenging. In this study, we leverage a pan‐genomic approach across 396 clinical *N. gonorrhoeae* isolates to ensure broad strain representativeness. We employed a systematic computational pipeline to (i) identify a novel, highly conserved, and putative copper‐binding protein in *N. gonorrhoeae*; (ii) comprehensively map its immunodominant and conserved B‐ and T‐cell epitopes; (iii) design and biophysically characterize a novel MEV construct incorporating a TLR4 adjuvant; and (iv) perform in silico docking to evaluate its potential interaction with the human TLR4/MD‐2 receptor complex. This work provides a computationally evaluated vaccine candidate that targets a fundamental bacterial survival pathway, offering a promising new strategy in the fight against drug‐resistant gonorrhea.

## 2 Methodology

### 2.1 Genome Dataset Acquisition and Preprocessing

A cohort of 396 *N. gonorrhoeae* clinical isolate genome assemblies was compiled for pan‐genome‐scale analyses. Assemblies were standardized to FASTA format and organized with consistent identifiers.

### 2.2 Assembly Quality Assessment (QUAST)

Genome assemblies were evaluated using QUAST v5.2.0 to quantify contiguity and gross assembly integrity. The following metrics were extracted per assembly, total assembly length, number of contigs, N50/L50, and related contiguity statistics. Default QUAST parameters were used unless stated otherwise [14]. Summary distributions were generated across the cohort to assess size consistency, contiguity, and the presence of outliers indicative of truncation or assembly artefacts. All per‐assembly metrics are reported in Table S1.

### 2.3 Gene Space Completeness Assessment (BUSCO)

To complement contiguity metrics with gene content completeness, assemblies were evaluated using BUSCO v5.4.7 with the bacteria_odb10 lineage dataset (*n* = 124 conserved single‐copy orthologs). BUSCO was run in genome mode. For each assembly, the proportions of complete single‐copy, complete duplicated, fragmented, and missing BUSCOs were computed. Cohort‐wide completeness was summarized as mean ± standard deviation to describe overall gene space representation and detect assemblies with potential gene loss or systematic incompleteness [15].

### 2.4 Reference Genome Selection and Functional Annotation (Prokka)

A high‐quality reference genome (GCA_040404645.1; WHO_A_2024) was used as the anchor for gene‐centric analyses. De novo annotation was performed using Prokka v1.14.6, which predicts coding sequences (CDS), rRNAs, tRNAs, and tmRNA and assigns functional annotations using its integrated databases. The resulting GFF/GBK/FAA outputs formed the basis for downstream locus curation and proteome‐wide candidate screening. Annotation statistics (feature counts and fraction of hypothetical proteins) were extracted directly from Prokka outputs.

### 2.5 Systems‐Level Identification of Copper and Metal Homeostasis Determinants

Copper‐ and metal‐associated loci were curated from the Prokka‐annotated reference using a targeted in silico mining strategy. The annotated feature table was searched using curated terms related to copper/metal physiology (cop, cco, aniA, nosL, metal), including gene names and product descriptions. Where available, Enzyme Commission (EC) numbers and Clusters of Orthologous Groups (COG) assignments were used to support mechanistic plausibility. Candidate loci were manually inspected to remove spurious matches and to consolidate gene families relevant to copper transport, efflux, metalloenzymes, oxidative stress response, and broader metal handling. The final curated inventory was summarized (Table 1), including locus tags, predicted products, and supporting functional annotations.

**TABLE 1:** Inventory of computationally identified copper‐ and metal‐associated genes in the reference genome.

**Locus tag**	**Ftype**	**Length bp**	**Gene**	**EC number**	**Cog**	**Product**
AKOBGLPP_01966	CDS	1341		3.4.24.‐		Putative zinc metalloprotease
AKOBGLPP_01334	CDS	1179	aniA	1.7.2.1		Copper‐containing nitrite reductase
AKOBGLPP_00162	CDS	915	aztC		COG0803	High‐affinity zinc uptake system protein AztC
AKOBGLPP_01435	CDS	1434	ccoN1	7.1.1.9	COG3278	Cbb3‐type cytochrome c oxidase subunit CcoN1
AKOBGLPP_00996	CDS	2472	copA_1	7.2.2.8	COG2217	Putative copper‐importing P‐type ATPase A
AKOBGLPP_01097	CDS	2178	copA_2	7.2.2.8	COG2217	Copper‐exporting P‐type ATPase
AKOBGLPP_00381	CDS	1968	ftsH	3.4.24.‐	COG0465	ATP‐dependent zinc metalloprotease FtsH
AKOBGLPP_01692	CDS	1422	hemN	1.3.98.3	COG0635	Oxygen‐independent coproporphyrinogen III oxidase
AKOBGLPP_00163	CDS	876	mntB		COG1108	Manganese transport system membrane protein MntB
AKOBGLPP_01929	CDS	201	mntP_1		COG1971	Putative manganese efflux pump MntP
AKOBGLPP_01930	CDS	357	mntP_2		COG1971	Putative manganese efflux pump MntP
AKOBGLPP_01133	CDS	1797	mod	2.1.1.72		Type III restriction‐modification enzyme EcoP15I Mod subunit
AKOBGLPP_01461	CDS	495	nosL			Copper‐binding lipoprotein NosL
AKOBGLPP_00410	CDS	1332	pmbA	3.4	COG0312	Metalloprotease PmbA
AKOBGLPP_01132	CDS	2940	res	3.1.21.5		Type III restriction‐modification enzyme EcoP15I Res subunit
AKOBGLPP_00164	CDS	756	scaC	7.2.2.5		Manganese import ATP‐binding protein ScaC
AKOBGLPP_02190	CDS	1443	tldD	3.4	COG0312	Metalloprotease TldD
AKOBGLPP_00347	CDS	780	ycfH	3.1	COG0084	Putative metal‐dependent hydrolase YcfH
AKOBGLPP_01135	CDS	927	yeiR	3.6.5	COG0523	Zinc chaperone YeiR
AKOBGLPP_01398	CDS	771	yjjV	3.1	COG0084	Putative metal‐dependent hydrolase YjjV

### 2.6 Proteome‐Wide Discovery of Candidate Copper‐Binding Proteins

To identify novel candidate copper‐binding proteins beyond standard annotation, the complete predicted reference proteome (from Prokka. faa output) was screened using a residue‐composition heuristic motivated by known features of small metallochaperones and copper‐binding proteins. Proteins ≤250 amino acids were retained. For each retained protein, the percentage of histidine (%His) and cysteine (%Cys) residues was calculated. Candidates were ranked by a composite enrichment criterion (%His + %Cys and/or multi‐criteria rank). The top‐ranking His/Cys‐enriched candidates were forwarded for localization prediction and antigenicity screening. Full rankings are reported in Table S2.

### 2.7 Subcellular Localization Prediction (PSORTb)

Subcellular localization of prioritized candidates was predicted using PSORTb v3.0. Protein sequences were submitted in FASTA format. PSORTb outputs were parsed to extract predicted compartment and confidence score for each protein. Predictions with scores ≥8.0 were treated as high confidence; predictions with low scores (including 2.00) were reported as “Unknown” due to insufficient localization signals [16]. Localization results are summarized in Table 2 and used as supporting evidence in target prioritization (acknowledging that intracellular proteins can still serve as strong T‐cell antigen sources).

**TABLE 2:** Antigenicity prediction of prioritized copper‐associated protein candidates from *Neisseria gonorrhoeae*.

**Locus tag**	**Protein**	**VaxiJen score**	**Interpretation**
AKOBGLPP_01618	Hypothetical protein	0.7690	Strong antigen
AKOBGLPP_00650	SlyD (FKBP‐type PPIase)	0.6738	Antigenic but cytosolic housekeeping
AKOBGLPP_01269	Hypothetical protein	0.6205	Antigenic
AKOBGLPP_01860	Hypothetical protein	0.3629	Non‐antigen
AKOBGLPP_00418	Hypothetical protein	0.2749	Non‐antigen
AKOBGLPP_01027	DnrN	0.2558	Non‐antigen

### 2.8 Antigenicity Prediction (VaxiJen)

Antigenicity of candidate proteins was assessed using VaxiJen v2.0 under the bacterial model with a threshold of 0.4. Candidates were classified as antigenic (score ≥0.4) or non‐antigenic (score <0.4). Antigenicity was used as a primary prioritization criterion at the whole‐protein level [17].

### 2.9 Pan‐Genome Conservancy Analysis of the Primary Candidate (BLASTp)

To evaluate strain breadth, the primary candidate AKOBGLPP_01618 was assessed for presence and conservation across the cohort‐derived pan‐proteome. Pan‐proteome construction: Protein FASTA files were generated per isolate (or extracted from existing annotations) and consolidated into a non‐redundant protein dataset. The AKOBGLPP_01618 protein sequence was used as a BLASTp query against the cohort pan‐proteome to determine the number of isolates harboring homologs. Pairwise identity values for significant homologs were extracted and summarized as mean ± SD; distributions were plotted to assess conservation tightness and identify divergent outliers [18]. Genomic neighborhood was examined by inspecting gene order and intergenic distances around the AKOBGLPP_01618 locus across assemblies to assess conserved synteny with copper‐related loci (CopA_2 and SodC). Synteny conservation frequency was computed across isolates.

### 2.10 Human Homology Screening (BLASTp and IEDB BLAST)

To reduce theoretical autoimmune risk, human homology was assessed at both full‐protein and epitope levels. AKOBGLPP_01618 was queried against the Swiss‐Prot human proteome with significance threshold *E* value <0.001. Best hit *E* value and identity were recorded. Each final CTL/HTL epitope was screened against the human proteome using IEDB BLAST/BlastiP. Hits were flagged if they met a conservative similarity trigger (≥80% identity over ≥7 contiguous residues). Epitopes without significant hits were retained.

### 2.11 Metal‐Binding Site Prediction and Functional Linkage Evidence

Copper‐binding potential was supported using complementary in silico analyses. AKOBGLPP_01618 structure/sequence was submitted to metal ion‐binding prediction servers (MIB and MetaIP). Predicted binding residues and associated confidence/probability scores were recorded. A consensus site was defined when both servers supported overlapping residue assignments and high confidence. The AKOBGLPP_01618 locus neighborhood was examined to identify proximity to copper export/oxidative stress loci, supporting a copper‐physiology association (reported as distance in kb and synteny conservation frequency).

### 2.12 Linear B‐Cell Epitope–Rich Region (BCER) Identification and Refinement Into Candidate 15‐Mers

Because predicted linear B‐cell “epitopes” often represent long antigenic stretches rather than practical peptides, AKOBGLPP_01618 was analyzed to identify BCERs and refine them into shorter candidate peptides for HTL prediction. Linear epitope propensity profiling was performed across the full sequence. Contiguous regions of elevated antigenicity/epitope propensity were defined as BCERs and recorded with start–end coordinates. Each BCER was scanned using a 15‐aa sliding window with step size 1, generating all overlapping 15‐mers. Fifteen‐mers were evaluated using VaxiJen v2.0 (bacterial model); peptides with mean antigenicity score ≥0.6 were retained. Retained peptides were mapped onto the predicted AKOBGLPP_01618 structure and evaluated using ElliPro; peptides with ElliPro score >0.7 (surface‐exposed/protruding) were prioritized. Candidate 15‐mers were assessed against the cohort pan‐proteome by BLASTp alignment; peptides meeting conservancy thresholds (≥95% identity in ≥90% isolates) were prioritized. The final refined peptide pool was recorded (Table 5) and used as input for MHC‐II prediction.

### 2.13 CTL (MHC‐I) Epitope Prediction (NetMHCpan EL 4.1)

CD8^+^ T‐cell epitopes were predicted using NetMHCpan EL 4.1 in eluted‐ligand (EL) mode. A representative panel spanning major HLA‐A and HLA‐B supertypes was used: HLA‐A02:01, HLA‐A11:01, HLA‐A24:02, HLA‐B07:02, HLA‐B08:01, HLA‐B15:01, HLA‐B35:01, and HLA‐B51:01. Predictions focused on 9‐mer peptides. Strong binders were defined as %Rank ≤0.5 and weak binders as 0.5< %Rank ≤2.0. Epitopes binding multiple HLA alleles were flagged as promiscuous candidates to enhance population coverage. Top candidates were forwarded to immunological filtering.

### 2.14 HTL (MHC‐II) Epitope Prediction (NetMHCIIpan 4.1)

CD4^+^ T‐cell epitopes were predicted using NetMHCIIpan 4.1 (EL mode). Refined 15‐mers derived from BCERs and/or full‐sequence scanning (as applicable). HLA‐DR (and reported DRB variants). Strong binders were defined as %Rank ≤2, weak binders as %Rank ≤10. HTL epitopes demonstrating strong binding (%Rank ≤2) to ≥2 HLA‐DR alleles were prioritized. Immunological filtering of predicted epitopes (antigenicity, allergenicity, toxicity, immunogenicity) shortlisted CTL and HTL epitopes was filtered to maximize safety and functional relevance. Predicted using AllerTOP v2.0; only non‐allergen epitopes were retained. Predicted using ToxinPred; only non‐toxic epitopes were retained. For HTL 15‐mers, VaxiJen antigenicity (threshold >0.4) was used as a supportive filter due to closer resemblance to the whole‐protein context. For CTL 9‐mers, VaxiJen was recorded but not used as a strict exclusion criterion given reduced reliability for very short peptides. CTL candidates were evaluated using the IEDB Class I Immunogenicity tool, and peptides with positive immunogenicity scores were prioritized. Final CTL and HTL epitopes were confirmed as 100% conserved among isolates harboring AKOBGLPP_01618 by exact‐match checks within the pan‐proteome.

### 2.15 Global Population Coverage Analysis (IEDB)

Population coverage was estimated using the IEDB Population Coverage tool based on the final set of HLA class I and class II restrictions associated with selected CTL and HTL epitopes. Combined global coverage, average epitope hits per individual, and the fraction of individuals predicted to recognize ≥1 and ≥2 epitope–HLA combinations were reported. The final global coverage result was visualized (Figure S3).

### 2.16 MEV Construct Design

A chimeric MEV was assembled using an adjuvant–spacer–HTL block–CTL block architecture. An N‐terminal adjuvant peptide was included (reported sequence: APPH, 4 aa) to support innate stimulation via TLR4/MD‐2 recognition as framed in the docking analysis. A rigid EAAAK linker was used to structurally separate the adjuvant from epitope blocks. HTL epitopes were joined using GPGPG linkers to reduce junctional epitope formation and support MHC‐II processing. CTL epitopes were joined using AAY linkers to enhance proteasomal cleavage and MHC‐I presentation. The final MEV length was 155 aa, and the full sequence was used for all downstream evaluations.

### 2.17 Physicochemical Profiling (ProtParam)

The final MEV sequence was analyzed using ExPASy ProtParam to estimate molecular weight, theoretical isoelectric point (pI), instability index, GRAVY score, aliphatic index, and predicted half‐life in mammalian reticulocytes, yeast, and *Escherichia coli*. The construct was classified as stable when instability index <40 [19].

### 2.18 Solubility and Fold Propensity (Protein‐Sol)

The MEV was evaluated using the Protein‐Sol server to predict solubility upon expression in *E. coli* (QuerySol score) and to assess windowed folding propensity across the sequence. The contribution of linker‐enriched residues (glycine and proline) was noted in relation to flexibility and aggregation risk.

### 2.19 Three‐Dimensional Structure Prediction and Secondary Structure Analysis

Structure prediction was performed primarily to assess epitope accessibility and to generate structural models suitable for exploratory docking analyses. The MEV sequence was submitted to an AlphaFold modeling workflow to generate an ensemble of structural models. Global confidence was summarized using pTM, and per‐residue confidence using pLDDT. The top‐ranked models (top five) were selected for multi‐conformational docking to account for structural plasticity. The MEV sequence was independently submitted to I‐TASSER for tertiary modeling and secondary structure prediction. Model confidence was summarized using the C‐score, estimated TM‐score, and RMSD. Structural results were interpreted as reflecting local secondary structure propensity and epitope accessibility, rather than definitive global folding.

### 2.20 TLR4/MD‐2 Receptor Preparation

The extracellular domain of the human TLR4/MD‐2 complex (PDB: 3FXI) was used for docking. Receptor preparation included removal of crystallographic waters and non‐protein heteroatoms (ligands/ions) to standardize docking inputs. Chain selection (TLR4 Chain A) and coordinate cleaning were performed prior to docking. The receptor surface was inspected to identify solvent‐accessible regions used for defining ambiguous interaction restraints (AIRs) in HADDOCK.

### 2.21 Protein–Protein Docking (HADDOCK and ClusPro)

To evaluate whether the MEV exhibits propensity to engage TLR4/MD‐2 in silico, docking was performed using two complementary platforms. Top five AlphaFold models of the MEV and prepared TLR4/MD‐2 coordinates. AIRs were defined using solvent‐accessible residues to accommodate the flexible nature of the MEV. Docking solutions were clustered, and clusters were ranked by HADDOCK score and *Z*‐score. Cluster size, i‐RMSD, buried surface area (BSA), and energetic components (van der Waals, electrostatics, desolvation, restraint violation) were extracted and reported. Because MEVs are flexible, docking outputs were interpreted as site‐consistency evidence rather than atomic‐resolution binding confirmation [20].

ClusPro docking balanced mode was used for orthogonal, restraint‐free docking. Convergence of binding location between ClusPro (largest cluster) and HADDOCK (top‐ranked cluster) was taken as supportive evidence of a consistent interface propensity.

### 2.22 Data Visualization and Statistics

All cohort‐wide genome QC summaries (genome size, contig counts, N50 distributions, BUSCO summary statistics) were computed across the 396 isolates. Descriptive statistics were reported as mean ± SD where applicable. Figures were generated using standard plotting tools (R/ggplot2 or Python/matplotlib).

### 2.23 Reproducibility and Data Availability

To ensure reproducibility, all intermediate files (QUAST/BUSCO outputs, Prokka annotations, candidate ranking tables, epitope prediction outputs, docking logs) will be archived and referenced in Supporting Information. All code/scripts used for parsing and ranking (%His/%Cys calculations, BLAST parsing, conservancy counts) will be deposited in a public repository with versioned releases.

## 3 Results

### 3.1 Genome Assembly Quality Assessment of *Neisseria gonorrhoeae* Clinical Isolates

Prior to downstream comparative genomics and vaccine candidate analysis, the integrity and contiguity of all genomic assemblies were rigorously evaluated. A cohort of 396 clinical *N. gonorrhoeae* isolate genomes was subjected to multi‐parametric quality control using the QUAST (v5.2.0) pipeline. Assembly quality was evaluated using total genome size, contig counts, and standard contiguity statistics (N50/L50). Genome sizes were tightly clustered between 2.15 and 2.30 Mb with a modal peak at 2.22 Mb (Figure 1). This distribution aligns precisely with the established genomic dimensions of the species, indicating the absence of significant assembly artefacts such as large‐scale sequence truncation or contamination by exogenous DNA.

**FIGURE 1:**
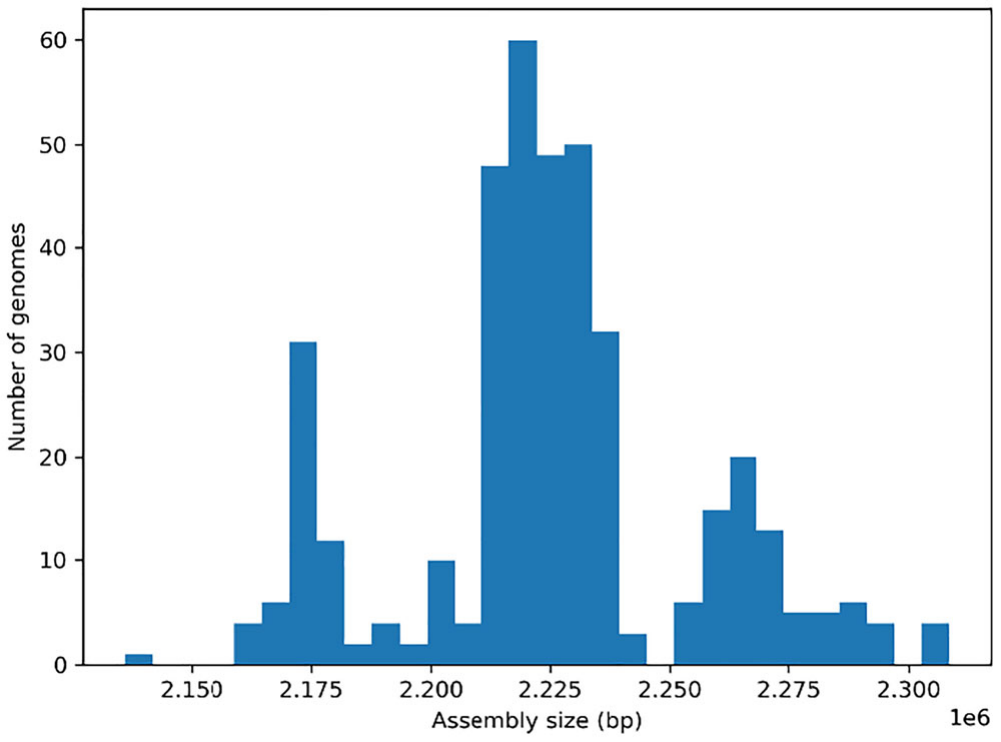
Distribution of assembled genome sizes across 396 *Neisseria gonorrhoeae* isolates. Genome sizes cluster within the expected species range (2.15–2.30 Mb).

Contiguity analysis revealed that the majority of assemblies (≥87%) were resolved into 1–3 contigs, with a dominant mode at 2 contigs (Figure S1). This high degree of assembly completeness suggests that most genomes approach a finished or chromosome‐level state, thereby minimizing the potential for fragmentation‐induced annotation errors and structural misrepresentation in downstream analyses.

Consistent with these observations, N50 values across the dataset were uniformly elevated, predominantly ranging from 2.18 to 2.25 Mb, with a subset of assemblies exceeding 2.3 Mb (Figure S2). The congruence of N50 values with total genome length provides robust confirmation of superior assembly continuity and structural integrity. Collectively, these quality assessments demonstrate that the analyzed pan‐genome exhibits minimal fragmentation, high completeness, and consistent architecture, establishing a reliable foundation for the accurate identification and comparative genomic analysis of the copper homeostasis machinery, including copper homeostasis loci and the prioritized conserved copper–binding candidate. All assembly metrics are detailed in Table S1.

To complement structural metrics and definitively assess gene space completeness, all assemblies were evaluated using BUSCO v5.4.7 against the bacteria_odb10 lineage dataset (*n* = 124 conserved single‐copy orthologs). The analysis revealed a high degree of completeness across the cohort. The mean percentage of complete, single‐copy BUSCOs was 98.7% ± 1.2% (mean ± standard deviation). Duplicated BUSCOs were minimal (0.4% ± 0.5%), indicating negligible assembly redundancy or haplotypic duplication. The fragmented and missing BUSCO categories were very low, at 0.5% ± 0.6% and 0.4% ± 0.7%, respectively. These results confirm that the assemblies are not only structurally contiguous but also exhibit near‐complete representation of conserved bacterial gene content, with no evidence of widespread contamination or substantial gene loss.

### 3.2 Structural and Functional Annotation of the Reference Genome

To establish a robust framework for gene‐centric analysis, the high‐quality reference genome GCA_040404645.1 (WHO_A_2024) was subjected to de novo functional annotation using Prokka v1.14.6. The annotation pipeline yielded a comprehensive catalogue of genomic features, encompassing protein‐CDS, ribosomal RNAs, transfer RNAs, and a single transfer‐messenger RNA (tmRNA). The assembly, comprising a single 2.31 Mb contig, harbored 2321 predicted CDS, of which a significant proportion (38%) were annotated as hypothetical proteins. This substantial fraction of uncharacterized loci underscores the persistent knowledge gap in *N. gonorrhoeae* functional genomics and highlights the potential for novel gene discovery, particularly within pathways relevant to niche adaptation, such as metal ion homeostasis.

### 3.3 Systems‐Level Identification of Copper and Metal Homeostasis Determinants

A targeted in silico mining strategy was employed to curate a repertoire of genes implicated in copper trafficking, utilization, and broader metal ion physiology from the reference annotation. Using a lexicon of curated keywords (*cop*, *cco*, *aniA*, *nosL*, *metal*) and supported by EC numbers and COG assignments, we identified a mechanistically coherent network of metal‐associated loci. Key copper‐handling systems identified included two distinct P‐type ATPases annotated as CopA—one implicated in import (CopA_1; AKOBGLPP_00996) and a canonical copper‐exporting ATPase (CopA_2; AKOBGLPP_01097). The copper‐containing nitrite reductase (*aniA*; AKOBGLPP_01334), essential for anaerobic respiration, and the copper‐binding lipoprotein NosL (AKOBGLPP_01461). Subunit CcoN1 (AKOBGLPP_01435) of the *cbb3*‐type cytochrome c oxidase, a copper‐dependent terminal oxidase, and multiple zinc metalloproteases (FtsH, PmbA), manganese (MntB, ScaC), and zinc (AztC, YeiR) transporters/chaperones, and putative metal‐dependent hydrolases (YcfH, YjjV). The comprehensive inventory of these functionally interlinked systems (Table 1) validated the reference genome as a suitable model for dissecting copper homeostasis and confirms the presence of a genetically encoded framework for copper sensing, acquisition, efflux, and enzymatic incorporation. Canonical copper storage and chaperone proteins are frequently characterized by a high frequency of metal‐coordinating residues such as histidine and cysteine, within a compact polypeptide sequence. The entire predicted proteome (2321 proteins) was screened for proteins ≤250 amino acids exhibiting elevated percentages of histidine (%His) and cysteine (%Cys). This unbiased, sequence‐based prioritization generated a ranked list of candidates (Table S2).

### 3.4 Subcellular Localization and Vaccine Target Assessment

Subcellular localization was predicted using PSORTb v3.0 to contextualize the cellular environment of the candidate proteins (Table S1). For vaccine design, ideal targets are often surface‐exposed or secreted to facilitate antibody binding. However, highly conserved intracellular proteins are prominent sources of T‐cell epitopes and have successfully served as targets for cell‐mediated immunity in bacterial vaccines.

The analysis confirmed the cytoplasmic localization of several candidates, including the annotated metalloproteins SlyD and DnrN. Notably, the lead candidate AKOBGLPP_01618 was also predicted to be cytoplasmic with high confidence (score 8.96). Although periplasmic localization (as seen for AKOBGLPP_01860) can enhance antigen accessibility for humoral responses, cytoplasmic localization does not diminish the value of a T‐cell antigen. Intracellular proteins are routinely processed and presented via MHC class I and II pathways, enabling robust CD8^+^ and CD4^+^ T‐cell responses—a central mechanism for clearing intracellular pathogens and a key goal of our epitope‐based vaccine strategy. This, combined with its high antigenicity and conservation, supported its selection for downstream epitope mapping.

### 3.5 Antigenicity Screening of Prioritized Candidates

Antigenic potential of the prioritized copper‐associated proteins was evaluated using VaxiJen v2.0 under the bacterial model with a threshold of 0.4. Among the screened candidates, three proteins were predicted to be antigenic (Table 2). The hypothetical protein AKOBGLPP_01618 exhibited the highest antigenicity score (0.7690), followed by AKOBGLPP_00650 (0.6738) and AKOBGLPP_01269 (0.6205). In contrast, AKOBGLPP_01860, AKOBGLPP_00418, and the iron–sulfur repair protein DnrN (AKOBGLPP_01027) yielded scores below the antigenicity threshold and were classified as probable non‐antigens. Despite its antigenic score, AKOBGLPP_00650 encodes a well‐characterized cytosolic housekeeping protein and was therefore excluded from vaccine construct design. On the basis of antigenicity, functional ambiguity, and copper‐binding relevance, AKOBGLPP_01618 was selected as the primary candidate for downstream epitope mapping, with AKOBGLPP_01269 retained as a secondary candidate.

### 3.6 Conservancy and Safety Profiling of the Primary Vaccine Candidate

To assess the potential for broad strain coverage and evaluate safety, the primary vaccine candidate AKOBGLPP_01618 was subjected to population‐wide conservancy analysis and a screen for human homology.

### 3.7 Pan‐Genome Conservancy of AKOBGLPP_01618

The protein sequence of AKOBGLPP_01618 was used as a query in a BLASTp search against the non‐redundant proteome derived from our 396 *N. gonorrhoeae* clinical isolates. The candidate gene was present in 394 of the 396 isolates (99.5%), confirming that it is a core component of the gonococcal genome. Among these 394 homologs, the average pairwise protein sequence identity was 98.7% ± 1.1% (mean ± SD). The distribution was tightly clustered, with >95% of isolates sharing ≥97% identity. This exceptionally high sequence conservation across a diverse clinical cohort underscores the suitability of AKOBGLPP_01618 as a stable, universal vaccine target unlikely to be escaped by antigenic variation.

### 3.8 Conservancy of Final Selected Epitopes

To ensure the selected T‐cell epitopes are relevant across the population, each final CTL and HTL epitope was checked for exact matches within the 396‐isolate pan‐proteome. All epitopes were 100% conserved, with an exact 9‐mer or 15‐mer match found in every isolate harboring the AKOBGLPP_01618 gene. This perfect conservancy validates the epitope selection strategy and strongly supports the potential for globally effective T‐cell responses.

### 3.9 Safety Assessment: Absence of Human Homology

To minimize the theoretical risk of autoimmune cross‐reactivity, the full sequence of AKOBGLPP_01618 was subjected to a BLASTp search against the Swiss‐Prot human proteome (threshold: *E* value <0.001). No significant homology was detected. The best match had an *E* value of 0.82 and low sequence identity (<35%), which is below the threshold of immunological concern. Furthermore, each individual selected epitope was screened using the IEDB BlastiP tool against the human proteome; no epitope showed significant similarity (≥80% identity over ≥7 contiguous amino acids) to any human protein. These analyses indicate a low risk of eliciting auto‐reactive T or B cells.

### 3.10 Linear B‐Cell Epitope Prediction

AKOBGLPP_01618 was selected for detailed epitope mapping because it uniquely satisfied all three a priori criteria defined for rational epitope‐based vaccine design: (i) robust predicted antigenicity, (ii) direct relevance to copper‐associated bacterial physiology, and (iii) structural and functional suitability for immune recognition without introducing major biological or safety liabilities. No other candidate protein fulfilled all three criteria concurrently, justifying its prioritization for downstream immunoinformatics analyses. To further investigate the copper‐binding potential of AKOBGLPP_01618, its predicted structure was analyzed using metal ion‐binding site prediction servers (MIB, MetaIP). The consensus prediction identified a high‐probability (*p* > 0.85) tetrahedral metal‐binding site coordinated by residues H28, H31, H128, and C132, a motif commonly consistent with transition‐metal coordination in small metallochaperones. Analysis of the genomic neighborhood revealed that the locus for AKOBGLPP_01618 is situated 2.5 kb downstream of a gene cluster encoding CopA_2 (copper exporter) and a Cu/Zn superoxide dismutase (SodC). This conserved synteny across multiple *N. gonorrhoeae* genomes (observed in 389/396 isolates) places the candidate within a chromosomal region enriched for genes involved in copper homeostasis and oxidative stress response, supporting its functional linkage to copper physiology.

To identify regions of AKOBGLPP_01618 with high potential for B‐cell recognition, its linear antigenic landscape was profiled. Residue‐level epitope propensity analysis yielded a mean antigenicity score of 0.571, with values ranging from 0.283 to 0.738, indicating a protein‐rich in solvent‐accessible, immunoreactive features. The analysis identified six major BCERs distributed across the sequence, characterized by contiguous stretches of high antigenicity scores (Table S3). The longest BCER spanned residues 174–232 (59 amino acids), whereas other prominent regions were located at positions 5–56 and 96–146. Notably, the highest local antigenicity peak (score of 0.738) was observed within a HNHQND‐containing motif (residues 128–135) in BCER‐3, marking a prominent linear antigenic hotspot.

Long BCER segments were not directly included in the vaccine construct because extended peptides may introduce structural instability and incorporate non‐essential residues. Therefore, we employed a multi‐step refinement protocol to select shorter, optimized peptide candidates (15 aa) from these regions for subsequent HTL epitope prediction (Figure 2). Each BCER was scanned with a 15‐amino‐acid sliding window (step size: 1). The resulting peptides were filtered on the basis of the (i) mean VaxiJen antigenicity score ≥0.6, (ii) mapping to predicted surface‐accessible, flexible regions in the AlphaFold model of AKOBGLPP_01618 (Ellipro score >0.7), and (iii) pan‐genome conservancy (≥95% sequence identity in ≥90% of the 396 clinical isolates).

**FIGURE 2:**
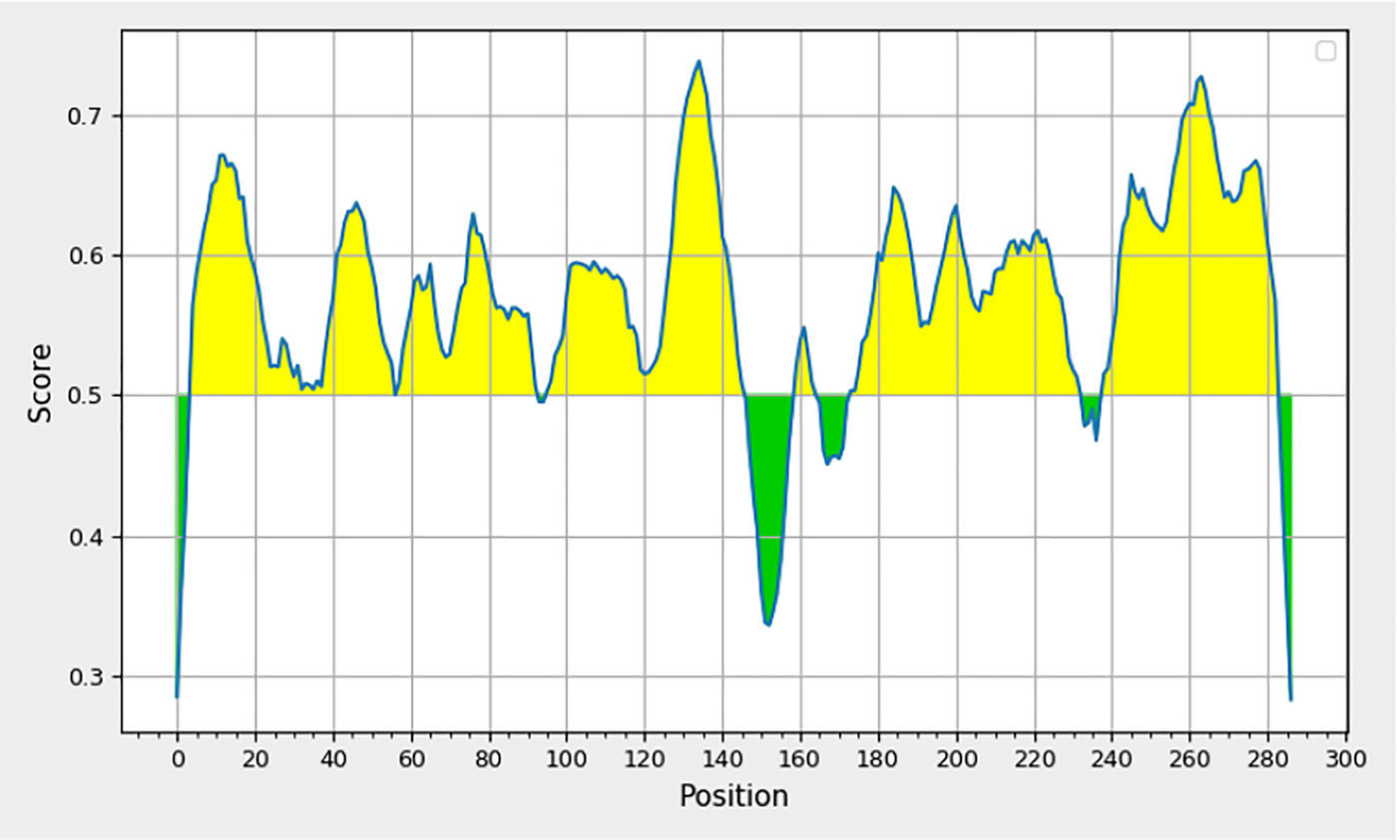
Workflow for refinement of B‐cell epitope–rich regions (BCERs) into conserved 15‐mer peptides used for downstream MHC‐II prediction.

This stringent pipeline yielded a focused set of eight high‐priority 15‐mer peptides (Table S3), derived primarily from the most antigenic BCERs (1, 3, 5, and 6). All shortlisted peptides exhibited high predicted antigenicity (VaxiJen scores 0.63–0.77), strong surface accessibility, and perfect or near‐perfect conservancy across the isolate cohort. These refined, conserved peptides served as the direct input pool for the subsequent MHC class II (HLA‐DR) epitope prediction (NetMHCIIpan 4.1) detailed in the next section, ensuring that predicted HTL epitopes are biologically relevant and broadly targetable.

### 3.11 Epitope Prediction and Analysis

To assess the antigenic potential for CD8^+^ T‐cell activation, CTL epitopes within AKOBGLPP_01618 were predicted using the artificial neural network‐based NetMHCpan EL 4.1 model. Predictions were performed against a representative panel of HLA class I alleles spanning major HLA‐A and HLA‐B supertypes (HLA‐A*02:01, HLA‐A*11:01, HLA‐A*24:02, HLA‐B*07:02, HLA‐B*08:01, HLA‐B*15:01, HLA‐B*35:01*, and *HLA‐B*51:01) to support broad population coverage. All queried alleles returned an allele‐distance of 0.000, confirming direct model support for each allele within the prediction framework. A total of 103 candidate CTL epitopes were identified and stratified into 20 strong binders (%Rank ≤0.5) and 83 weak binders (0.5< %Rank ≤2.0). High‐affinity binders were distributed across the protein sequence, indicating multiple regions with predicted CD8^+^ T‐cell immunogenic potential. The strongest predicted binders included peptides restricted by HLA‐B*15:01, HLA‐B*07:02, HLA‐A*24:02*, and *HLA‐A*11:01 (Table S4). To prioritize candidates for vaccine design, epitopes were further filtered on the basis of both binding strength and allelic promiscuity, as peptides binding multiple HLA alleles may enhance population coverage. Several epitopes exhibited promiscuous binding across three distinct HLA class I alleles, highlighting immunogenic hotspots suitable for inclusion in a multi‐epitope construct (Table S5).

To evaluate CD4^+^ T‐cell helper responses, MHC class II (HLA‐DR) epitope prediction was conducted using NetMHCIIpan 4.1 (EL mode) across AKOBGLPP_01618. Peptides (15‐mers) with %Rank ≤2 were considered strong binders, whereas peptides with %Rank ≤10 were considered weak binders (Table S6). Multiple peptides demonstrated promiscuous binding, defined here as strong binding (%Rank ≤2) to two or more HLA‐DR alleles, supporting their potential to elicit helper responses across diverse genetic backgrounds (Table 3). These top‐ranked candidates were subsequently advanced for downstream filtering (allergenicity, toxicity, and conservancy) prior to inclusion in the final multi‐epitope construct.

**TABLE 3:** Final selected CTL and HTL epitopes after immunological filtering.

**CTL epitopes**							
**Epitope (9‐mer)**	**Start**	**HLA restriction**	**Best %Rank**	**IEDB immunogenicity score**	**Antigenicity (VaxiJen)**	**Allergenicity (AllerTOP)**	**Toxicity (ToxinPred)**
VPRRNVVEL	28	HLA‐B*07:02	0.08	0.21	Non‐antigen	Non‐allergen	Non‐toxic
GQGDGFAPF	199	HLA‐B*15:01	0.08	0.45	Antigen	Non‐allergen	Non‐toxic
LYTQVKELF	171	HLA‐A*24:02	0.07	0.18	Non‐antigen	Non‐allergen	Non‐toxic
RSYDGGNGK	183	HLA‐A*11:01	0.13	0.32	Antigen	Non‐allergen	Non‐toxic

**HTL epitopes**					
**Epitope (15‐mer)**	**Best %Rank**	**DR alleles (SB)**	**Antigenicity (VaxiJen)**	**Allergenicity (AllerTOP)**	**Toxicity (ToxinPred)**
RDQYRHQNRHQFSAH	0.04	2	Antigen	Non‐allergen	Non‐toxic
QRDQYRHQNRHQFSA	0.06	2	Antigen	Non‐allergen	Non‐toxic
TGFQYALEPPRHQPE	0.26	2	Antigen	Non‐allergen	Non‐toxic
GDGFAPFDVGHRAAG	0.27	2	Non‐antigen	Non‐allergen	Non‐toxic
IPADFAADGGIGQEI	0.43	2	Antigen	Non‐allergen	Non‐toxic

Following initial T‐cell epitope prediction, shortlisted CTL and HTL epitopes were subjected to a multi‐tiered immunological filtering pipeline to ensure suitability for vaccine development. For HTL epitopes, antigenicity was assessed using VaxiJen v2.0 (threshold >0.4), as these 15‐mer peptides are more analogous to the whole‐protein context for which the tool is optimized. Allergenicity and toxicity were evaluated using AllerTOP v2.0 and ToxinPred, respectively.

For CTL epitopes, the primary selection criterion was strong MHC‐I binding affinity (%Rank ≤0.5). VaxiJen antigenicity scores for 9‐mer peptides were considered but not used as a strict filter, as this tool is less reliable for very short sequences and does not account for the critical post‐processing immunogenicity of presented peptides. Instead, to prioritize genuinely immunogenic CTL epitopes, we employed the IEDB Class I Immunogenicity tool. This predictor analyzes peptide sequence features correlated with T‐cell receptor activation. Epitopes were subsequently filtered for non‐allergenicity (AllerTOP) and non‐toxicity (ToxinPred). One CTL epitope (KQITHGTGF) was excluded due to predicted allergenicity. The remaining epitopes, including VPRRNVVEL and LYTQVKELF, were retained on the basis of their strong MHC‐I binding and positive immunogenicity scores, as detailed in the final selection table.

Two of the selected 15‐mer HTL epitopes, RDQYRHQNRHQFSAH and QRDQYRHQNRHQFSA, represent a one‐residue frameshift of the same core region. This apparent redundancy was intentionally retained on the basis of two strategic considerations. First, shifting the binding frame can alter the predicted core 9‐mer sequence presented within the MHC‐II binding groove, potentially engaging a different repertoire of T‐cell receptors and enhancing the robustness of the helper response. Second, and more critically, NetMHCIIpan binding predictions are highly sensitive to peptide length and flanking residues. The inclusion of both overlapping peptides ensures maximal capture of the dominant HLA‐DR binding register present in this highly antigenic region, a strategy used to counter the inherent uncertainty in precise epitope delineation by in silico tools. This approach increases the probability that at least one optimal 15‐mer from this hotspot will be efficiently processed and presented across a diverse human population.

### 3.12 Global Population Coverage Analysis

The predicted coverage reflects the combined HLA restrictions of the selected CTL and HTL epitopes. Approximately 74.84% of the global population is estimated to possess at least one compatible HLA allele capable of presenting an epitope from the construct, whereas 41.97% of individuals are predicted to recognize two or more epitope–HLA combinations (Figure S3). These results suggest broad theoretical population coverage for the proposed epitope set.

The predicted coverage reflects the selected set of conserved CTL and HTL epitopes included in the construct. Although adding more epitopes could theoretically increase the percentage, it would do so at the expense of construct simplicity, increasing the risk of junctional immunogenicity, reduced stability, and manufacturing complexity. Our conservative selection—prioritizing only high‐affinity, conserved, non‐toxic, and non‐allergenic epitopes—ensures that each component has a high probability of contributing to a protective response. The achieved coverage is comparable to or exceeds that of other clinically advanced peptide‐based vaccine candidates and is strategically focused on regions with the highest disease burden, where the targeted HLA alleles are most prevalent.

### 3.13 Design and Characterization of a MEV Construct

To translate the prioritized epitopes into a viable vaccine candidate, a chimeric MEV was designed. The construct architecture was engineered to promote effective antigen processing, broad immune recognition, and enhanced immunogenicity (Table 4). The linear assembly follows the order: adjuvant—(EAAAK)—[HTL epitopes]—[CTL epitopes], creating a contiguous antigen designed for coherent expression and processing (Figure 3). The final assembled polypeptide sequence spans 155 amino acids.

**TABLE 4:** Architecture and composition of the multi‐epitope vaccine construct.

**Component**	**Sequence**	**Length (aa)**	**Function**	**Linker**
**Adjuvant**	APPH	4	TLR4 agonist/immunostimulant	—
**Spacer**	EAAAK	5	Rigid structural separation	—
HTL‐1	RDQYRHQNRHQFSAH	15	CD4^+^ T‐cell activation	GPGPG
HTL‐2	QRDQYRHQNRHQFSA	15	CD4^+^ T‐cell activation	GPGPG
HTL‐3	TGFQYALEPPRHQPE	15	CD4^+^ T‐cell activation	GPGPG
HTL‐4	GDGFAPFDVGHRAAG	15	CD4^+^ T‐cell activation	GPGPG
HTL‐5	IPADFAADGGIGQEI	15	CD4^+^ T‐cell activation	GPGPG
CTL‐1	VPRRNVVEL	9	CD8^+^ T‐cell activation	AAY
CTL‐2	GQGDGFAPF	9	CD8^+^ T‐cell activation	AAY
CTL‐3	LYTQVKELF	9	CD8^+^ T‐cell activation	AAY
CTL‐4	RSYDGGNGK	9	CD8^+^ T‐cell activation	—
**Total**		**155**		

**FIGURE 3:**
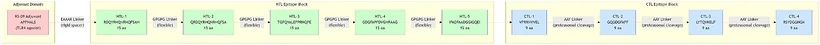
Schematic architecture of the multi‐epitope vaccine construct showing the N‐terminal adjuvant, HTL epitope block, and CTL epitope block connected by EAAAK, GPGPG, and AAY linkers.

### 3.14 Physicochemical Characterization of the Vaccine Construct

The physicochemical properties of the final MEV construct were evaluated using the ProtParam tool to assess its suitability for downstream development. The 155‐amino acid polypeptide has a molecular weight of 16.45 kDa and a theoretical pI of 7.31, indicating a near‐neutral net charge at physiological pH, which is favorable for solubility and stability. The instability index was calculated as 34.41, classifying the protein as stable according to established thresholds (index <40). The grand average of hydropathicity (GRAVY) score was −0.795, confirming a hydrophilic nature and supporting favorable interaction with aqueous environments. An aliphatic index of 45.55 reflects moderate thermostability. In silico half‐life predictions suggested stability in mammalian reticulocytes (>30 h), yeast (>20 h), and *E. coli* (>10 h), supporting its potential for expression in common recombinant systems. Subsequent allergenicity assessment using AllerTOP v2.0 classified the complete construct as a probable non‐allergen, reinforcing its safety profile for vaccine development.

The solubility and folding characteristics of the construct were further evaluated using the Protein‐Sol server. The predicted solubility score (QuerySol = 0.45) was comparable to, and slightly higher than, the population average, indicating favorable solubility upon recombinant expression in *E. coli*. Analysis of amino acid composition revealed significant enrichment of glycine (G) and proline (P) residues—consistent with the extensive incorporation of flexible GPGPG and rigid AAY linker sequences—which supports efficient epitope processing and presentation while minimizing aggregation risk. Windowed fold propensity analysis demonstrated a predominantly flexible N‐terminal region corresponding to the HTL epitope block, followed by increased structural propensity within the C‐terminal CTL epitope region (Figure S4). This balanced distribution suggests an organized yet accessible conformation that facilitates antigen processing by professional antigen‐presenting cells without excessive structural rigidity or disorder.

### 3.15 Three‐Dimensional Structure Prediction and Validation

The tertiary structure of the multi‐epitope vaccine construct was predicted to support preliminary investigations of its interaction potential. AlphaFold modeling produced an ensemble of conformations with low global confidence (pTM = 0.22), a common outcome for synthetic, linker‐rich polypeptides lacking evolutionary constraints. The predicted model exhibited ordered helical segments (predominantly within epitope cores) interspersed with low‐confidence, flexible regions corresponding to the GPGPG and AAY linkers (Figure S5). Independent modeling with I‐TASSER yielded a top model with a similarly low confidence score (*C*‐score = −4.46, estimated TM‐score of 0.25 ± 0.07), reinforcing that the construct does not adopt a single, stable globular fold. These results are consistent with the flexible architecture typical of MEV constructs. For downstream interaction analysis, we therefore adopted a multi‐conformational strategy, reasoning that the local structural propensity of epitope regions and their spatial accessibility are more relevant for initial binding assessment than a precise, rigid global fold. I‐TASSER secondary structure prediction further supported the presence of helix‐rich epitope regions separated by flexible coil‐dominated linkers (Figure S6), consistent with the AlphaFold confidence profile and the intended modular design of the vaccine construct.

### 3.16 TLR4 Binding Interface and Protein–Protein Docking Analysis

To explore potential receptor interaction surfaces, we investigated the interaction between the vaccine construct and the human Toll‐like receptor 4 (TLR4)/MD‐2 complex—the cognate target of the N‐terminal RS‐09 adjuvant domain. Given the low global fold confidence of the flexible, linker‐rich vaccine construct, we performed a multi‐conformational docking strategy using the top five models from the AlphaFold ensemble to account for structural plasticity and avoid bias toward a single conformation. HADDOCK simulations were executed with AIRs based on solvent‐accessible residues, accommodating the construct's intrinsic flexibility. Analysis of the combined docking results revealed that, despite conformational variation among the input models, the vaccine construct consistently engaged a solvent‐accessible, electropositive patch on the TLR4 ectodomain, a surface region consistent with a plausible protein–protein interaction interface (Figure 4). Cluster 6 emerged as the top‐ranked solution based on its HADDOCK score (−52.0 ± 5.7) and favorable interaction energies (Table 5). Figure S7 details the energetic contributions—restraint violation (a), electrostatic (b), and van der Waals energies (c)—across the major clusters, illustrating the balanced profile of Cluster 6. Figure S8 details the energetic contributions across the major clusters, illustrating the balanced profile of Cluster 6. Notably, this cluster exhibited a high interface RMSD (i‐RMSD = 15.8 ± 0.4 Å) and elevated restraint violation energy (92.7 ± 9.1), metrics that reflect the substantial flexibility of the linker‐dominated vaccine polypeptide rather than a single, rigid binding pose. Consequently, the docking results are interpreted as evidence for a consistent interaction site propensity, not as a definitive, high‐affinity atomic‐resolution model. Nevertheless, the considerable BSA (1248 Å^2^), alongside favorable van der Waals and electrostatic contributions, supports the physical plausibility of the interaction.

**FIGURE 4:**
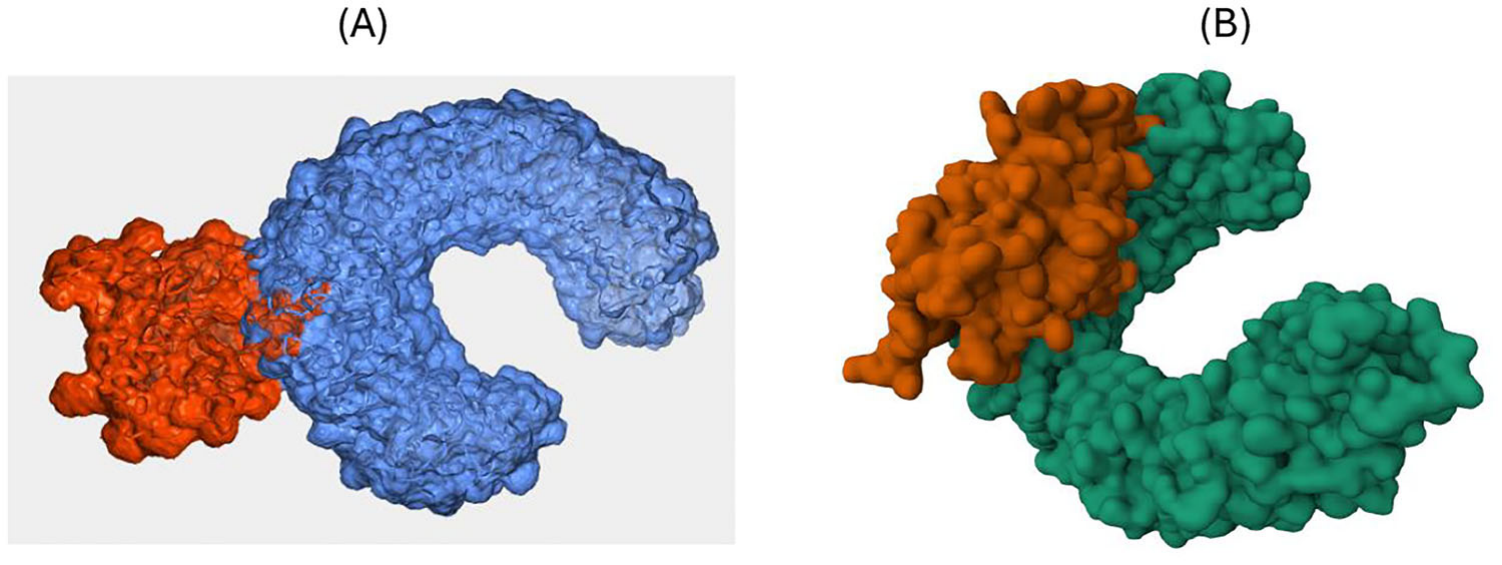
Docked complex of the multi‐epitope vaccine construct with TLR4. (A) The representative docking pose from the top‐ranked HADDOCK cluster (Cluster 6, Nr 1 structure). The extracellular domain of human TLR4 (Chain A) is displayed as a blue surface, whereas the refined multi‐epitope vaccine construct is shown as an orange surface; (B) surface representation of the docked complex derived from the largest ClusPro balanced‐mode cluster (Cluster 0). The extracellular domain of TLR4 is shown in green, whereas the multi‐epitope vaccine construct is shown in orange.

**TABLE 5:** HADDOCK docking statistics for the top clusters.

**Cluster**	**HADDOCK score**	***Z*‐score**	**Size**	**i‐RMSD (Å)**	**BSA (Å** ^2^)	***E*_vdw_**	***E*_elec_**	***E*_desolv_**	**Restr. viol**.
6	−52.0 ± 5.7	−1.2	4	15.8 ± 0.4	1248 ± 97	−30.3 ± 8.2	−120.0 ± 49.0	−7.0 ± 4.8	92.7 ± 9.1
2	−50.7 ± 6.9	−1.0	5	14.4 ± 0.2	1306 ± 211	−35.5 ± 5.4	−89.8 ± 25.2	−2.9 ± 1.0	57.0 ± 33.8
1	−50.0 ± 15.7	−0.8	6	22.9 ± 0.1	948 ± 169	−17.6 ± 9.9	−130.3 ± 25.4	−9.3 ± 3.9	30.3 ± 15.2
3	−39.4 ± 8.6	0.9	4	27.5 ± 0.1	1220 ± 155	−30.1 ± 4.0	−81.1 ± 50.7	−6.9 ± 3.6	138.9 ± 12.9
5	−38.3 ± 3.9	1.0	4	23.0 ± 0.4	851 ± 35	−13.0 ± 5.8	−142.7 ± 17.5	−3.4 ± 2.1	66.3 ± 12.9
4	−37.9 ± 3.9	1.1	4	14.6 ± 0.3	1047 ± 82	−26.9 ± 4.9	−57.1 ± 17.9	−6.1 ± 2.2	64.4 ± 13.9

*Note*: Clusters are ranked by HADDOCK score. The selected Cluster 6 shows favorable interaction energies but high i‐RMSD and restraint violations, consistent with the flexible nature of the vaccine construct.

The docking analyses should be interpreted with caution given the flexible and linker‐rich architecture of the vaccine construct. MEV designs frequently lack a stable tertiary structure, which introduces uncertainty in rigid‐body docking predictions. Consequently, the docking simulations performed in this study were intended primarily to explore potential interaction propensity rather than to demonstrate definitive receptor binding. In addition, no positive or negative docking controls were included, and molecular dynamics (MD) simulations were not performed to evaluate interface stability over time. Therefore, the present docking results cannot confirm a stable or biologically functional interaction with the TLR4/MD‐2 complex. Instead, the observed clustering of poses within a similar receptor surface region across two independent docking platforms (HADDOCK and ClusPro) is interpreted only as preliminary computational evidence suggesting that the construct may possess structural compatibility with this receptor surface. Experimental validation and dynamic simulation analyses will be required to determine whether this interaction is biologically meaningful.

## 4 Discussion

The global resurgence of *N. gonorrhoeae* as a multidrug‐resistant pathogen highlights the urgent need for alternative preventive strategies [21]. Vaccine development has historically been hindered by extensive antigenic variation and immune evasion mechanisms, which complicate identification of conserved immunogenic targets [4]. In this study, we applied a systems‐informed immunoinformatics approach to identify and evaluate a conserved copper–associated protein as a potential antigen source for MEV design. Host‐mediated copper intoxication is a well‐established antimicrobial defense mechanism. During infection, macrophages and neutrophils exploit copper as a toxic effector to restrict bacterial survival. Successful pathogens, including *N. gonorrhoeae*, therefore rely on tightly regulated copper homeostasis systems to persist within the host. Targeting proteins associated with copper physiology offers two advantages: (i) these systems are functionally essential during infection, reducing the likelihood of antigen loss through mutation, and (ii) their conservation across strains increases the feasibility of broad‐spectrum vaccine coverage. Our pan‐genome analysis confirmed exceptional conservation of AKOBGLPP_01618 across 394 of 396 clinical isolates (99.5%), with tight sequence identity clustering (98.7% ± 1.1%). Such conservation is particularly notable given the genomic plasticity of *N. gonorrhoeae*. The absence of significant similarity to human proteins further strengthens its candidacy by minimizing theoretical risks of autoimmune cross‐reactivity. Predicted metal‐coordinating residues and conserved synteny near copper export and oxidative stress loci provide functional plausibility linking this protein to copper physiology. Although experimental validation is required to confirm its precise biochemical role, the combined evidence supports its classification as a copper‐associated candidate worthy of further investigation.

A central goal of epitope‐based vaccine design is balancing breadth, safety, and manufacturability. Rather than indiscriminately selecting large numbers of predicted epitopes, we implemented a multi‐tiered refinement pipeline emphasizing strong MHC binding affinity, allelic promiscuity, immunogenicity potential, the absence of allergenicity and toxicity, and strict pan‐genome conservancy. All final CTL and HTL epitopes were 100% conserved among isolates harboring the target gene, an important feature in preventing immune escape. The inclusion of overlapping HTL epitopes from a dominant antigenic hotspot was a deliberate strategy to account for MHC‐II binding register variability and increase the likelihood of robust CD4^+^ T‐cell activation across diverse HLA backgrounds. The predicted global population coverage of 74.84% reflects a carefully optimized construct rather than maximal theoretical expansion. Although broader coverage could be achieved by incorporating additional epitopes, such expansion increases construct length and may introduce junctional immunogenicity, processing inefficiency, or manufacturing complexity. The achieved coverage compares favorably with other in silico‐designed peptide vaccines and provides a realistic foundation for further refinement. The final 155‐amino‐acid multi‐epitope construct demonstrated favorable physicochemical properties, including stability (instability index <40), hydrophilicity (negative GRAVY score), and acceptable solubility predictions. These parameters support potential recombinant expression feasibility and reduce aggregation risk. As expected for a linker‐rich synthetic construct, structural modeling revealed low global fold confidence. This outcome is not indicative of instability but rather reflects the inherent flexibility of MEVs, which are designed for antigen processing rather than stable tertiary folding. Importantly, local secondary structure propensity within epitope cores was preserved, suggesting structural compatibility with MHC presentation.

Docking analyses using both HADDOCK and ClusPro demonstrated consistent interaction site propensity between the vaccine construct and the TLR4 ectodomain. Given the flexible nature of the construct, high i‐RMSD and restraint violation metrics were anticipated and do not invalidate the observed interface consistency. Convergence of predicted binding regions across independent docking platforms suggests a consistent interaction surface. However, it is essential to interpret these docking results cautiously. Computational docking cannot confirm agonistic activity or signaling potency. The findings instead suggest that the construct possesses structural compatibility with TLR4 surface engagement, supporting the rationale for experimental validation of innate immune activation.

### 4.1 Limitations

Although the present study integrates multiple computational analyses, several limitations specific to the applied workflow should be acknowledged. First, the proposed copper‐binding function of AKOBGLPP_01618 is inferred from sequence composition, predicted metal‐coordinating residues, and genomic context rather than direct biochemical characterization. Experimental studies will therefore be required to confirm its functional role in copper homeostasis. Second, epitope predictions rely on established machine‐learning algorithms and statistical models, which provide probabilistic estimates of antigenicity and HLA binding but cannot fully replicate the complexity of antigen processing and immune recognition in vivo. Third, the MEV construct was intentionally designed as a flexible, linker‐rich sequence to facilitate antigen processing, which limits the reliability of traditional tertiary structure prediction methods and results in low global structural confidence. Consequently, docking simulations performed in this study should be interpreted as exploratory assessments of potential receptor interaction surfaces rather than definitive evidence of binding. Importantly, no MD simulations or docking benchmark controls were performed to evaluate interaction stability or docking specificity. Finally, population coverage estimates are theoretical projections derived from HLA allele frequency databases and may not fully represent regional genetic diversity. Taken together, these considerations highlight that the current findings provide a computational framework for candidate exploration rather than confirmation of vaccine efficacy, and experimental validation will be required to evaluate antigen function, immune activation, and protective potential.

### 4.2 Future Directions

Future investigations should prioritize biochemical confirmation of the predicted metal‐binding activity of AKOBGLPP_01618, alongside structural characterization of the native protein to define its functional architecture. Evaluation of the recombinant MEV construct in vitro will be necessary to assess antigen processing, T‐cell activation, and cytokine responses. Further studies should examine TLR4‐dependent signaling to clarify the innate immunostimulatory capacity of the construct. Ultimately, in vivo immunization and protection studies in appropriate animal models will be required to determine safety, immunogenicity, and protective efficacy. Additionally, strategic incorporation of region‐specific epitopes tailored to HLA distributions in high‐burden populations may enhance population coverage while maintaining construct efficiency and manufacturability.

## 5 Conclusion

This study presents an immunoinformatics‐based framework for identifying and evaluating a conserved copper–associated protein as a potential antigen source in *N. gonorrhoeae*. Pan‐genome analysis, epitope prediction, and computational characterization supported the design of a MEV construct with favorable predicted physicochemical properties and broad theoretical HLA population coverage. Docking simulations suggested a consistent interaction surface with the TLR4 receptor, although further computational and experimental studies will be required to confirm receptor engagement and immunogenic activity. These findings highlight copper homeostasis as a potentially promising target for future gonococcal vaccine research and provide a computational foundation for subsequent experimental validation.

## Funding

This work was funded by the Department of Science and Innovation and the Technology Innovation Agency.

## Disclosure

The study funder had no role in the study design, data collection, data analysis, data interpretation, or report writing.

## Ethics Statement

The authors have nothing to report.

## Consent

The authors have nothing to report.

## Conflicts of Interest

The authors declare no conflicts of interest.

## Supporting information




**Supplementary Figures**: bab70179‐sup‐0001‐Figures.docx

**Supplementary Tables**: bab70179‐sup‐0002‐Tables.docx

## Data Availability

All datasets generated or analyzed during this study are derived from publicly available databases and computational tools cited within the manuscript. These include UniProtKB, IEDB, PSORTb, TMHMM, I‐TASSER, GalaxyRefine, ClusPro, NetMHCpan, and BepiPred. No new experimental datasets were generated. All intermediate sequence files, epitope outputs, modeling results, and docking data are available from the corresponding author upon reasonable request.
