# Supplementary material for: Immunoinformatics‐Based Design of a Multi‐Epitope Vaccine Targeting a Conserved Copper–Associated Protein in Neisseria gonorrhoeae
Source: Biotechnol Appl Biochem. 2026 May 8;73(5):2373–87. doi: 10.1002/bab.70179 (PMC13644377; doi:10.1002/bab.70179)
Supplement: Supplementary file 1 [file BAB-73-2373-s001.docx]

**Supplementary Figures**

Figure S1: Histogram depicting the distribution of contig counts per assembly. The preponderance of low contig numbers signifies excellent assembly continuity, approaching near-complete chromosomal reconstruction.

Figure S2: Distribution of assembly N50 values. High N50 magnitudes, closely approximating total genome size, confirm excellent scaffold contiguity and the absence of pervasive fragmentation.

Figure S3: Global population coverage analysis of the selected CTL and HTL epitopes. Population coverage was estimated using the IEDB population coverage tool based on associated HLA class I and class II alleles.

Figure S4: Predicted solubility and fold propensity profile of the multi-epitope vaccine construct.

Figure S5: Predicted 3D structure of the multi‑epitope vaccine construct generated by AlphaFold. Colouring indicates per‑residue confidence (pLDDT). Low confidence (pLDDT < 50, orange) dominates linker‑rich segments, while epitope regions show higher local confidence (blue), reflecting their inherent secondary structure propensity.

Figure S6: I-TASSER-predicted secondary structure profile along the construct sequence, showing helix-enriched epitope regions interspersed with coil-dominated flexible linker segments. Helices (orange) correspond to epitope-rich segments, not linkers; Coils (grey) dominant across the central regions - linker-heavy segments (GPGPG, AAY).

(A)	(B)	(C)

Figure S7: Energetic characterization of docking clusters. (A) restraint violation energy; (B) electrostatic energy; and (C) van der Waals interactions.

Figure S8: HADDOCK cluster analysis (score vs i-RMSD, energy distributions).
