# Supplementary material for: Immunoinformatics‐Based Design of a Multi‐Epitope Vaccine Targeting a Conserved Copper–Associated Protein in Neisseria gonorrhoeae
Source: Biotechnol Appl Biochem. 2026 May 8;73(5):2373–87. doi: 10.1002/bab.70179 (PMC13644377; doi:10.1002/bab.70179)
Supplement: Supplementary file 2 [file BAB-73-2373-s002.docx]

**Supplementary Tables**

Table S1: Predicted subcellular localization of prioritized candidates.

| **Locus tag** | **Annotation** | **Predicted Localization** | **PSORTb Confidence Score** |
| --- | --- | --- | --- |
| AKOBGLPP_00650 | FKBP-type PPIase (SlyD) | Cytoplasmic | 9.97 |
| AKOBGLPP_01027 | DnrN (Fe–S repair protein) | Cytoplasmic | 9.97 |
| AKOBGLPP_01618 | Hypothetical protein | Cytoplasmic | 8.96 |
| AKOBGLPP_01860 | Hypothetical protein | Periplasmic | 9.83 |
| AKOBGLPP_00418 | Hypothetical protein | Unknown | 2.00 |
| AKOBGLPP_01269 | Hypothetical protein | Unknown | 2.00 |
| AKOBGLPP_01561 | Hypothetical protein | Unknown | 2.00 |
| AKOBGLPP_01626 | Hypothetical protein | Unknown | 2.00 |
| AKOBGLPP_01682 | Hypothetical protein | Unknown | 2.00 |
| AKOBGLPP_01889 | Hypothetical protein | Unknown | 2.00 |

*PSORTb scores ≥ 8.0 indicate high-confidence predictions. A score of 2.00 denotes insufficient localization signals ("Unknown").

Table S2: Predicted B-cell epitope–rich regions (BCERs) in AKOBGLPP_01618.

| **No.** | **Start–End** | **Length (aa)** | **Sequence (Representative Segment)** |
| --- | --- | --- | --- |
| 1 | 5–56 | 52 | FKPPRGNAAEKHHRDDVECKHPCVPVRRNVVELPVHEGRFGQKGEHRTEAEC |
| 2 | 58–93 | 36 | KKQITHRGTGFQYALEPPRHQPEHIPADFAADGGIG |
| 3 | 96–146 | 51 | ILYQTGQHRRIRRQENENRPPSAQSLQQCADHGSGQRHNHQNDADKGKHLR |
| 4 | 160–164 | 5 | DDDYS |
| 5 | 174–232 | 59 | TQVKELFGTRSKYDGGNGKAIYNQTGQGDGFAPFDVGHRAAGHLPERRAEHKQTQTLFD |

Table S3: Refined 15-mer peptides derived from B-cell epitope–rich regions (BCERs).

| **Peptide Sequence** | **Source BCER** | **VaxiJen Score** | **Conservancy (% isolates, ≥95% ID)** | **Surface Access (Ellipro Score)** |
| --- | --- | --- | --- | --- |
| KHPCVPVRRNVVELPV | 1 | 0.72 | 100% | 0.85 |
| HHRDDVECKHPCVPV | 1 | 0.70 | 100% | 0.81 |
| QRHNHQNDADKGKHL | 3 | 0.77 | 100% | 0.89 |
| HQNRHQFSAHRRKGR | 6 | 0.75 | 100% | 0.92 |
| GQGDGFAPFDVGHRA | 5 | 0.68 | 100% | 0.78 |
| DQYRHQNRHQFSAHR | 6 | 0.73 | 100% | 0.88 |
| RDQYRHQNRHQFSAH | 6 | 0.71 | 100% | 0.90 |
| QTGQGDGFAPFDVGH | 5 | 0.63 | 99.2% | 0.75 |

Table S4: High-affinity cytotoxic T-lymphocyte (CTL) epitopes predicted for AKOBGLPP_01618.

| **Epitope (9-mer core)** | **Start position** | **Best %Rank** | **EL score** | **Restricting HLA allele** |
| --- | --- | --- | --- | --- |
| KQITHGTGF | 59 | 0.05 | 0.84 | HLA-B*15:01 |
| VPRRNVVEL | 28 | 0.08 | 0.80 | HLA-B*07:02 |
| GQGDGFAPF | 199 | 0.08 | 0.79 | HLA-B*15:01 |
| LYTQVKELF | 171 | 0.07 | 0.75 | HLA-A*24:02 |
| RSYDGGNGK | 183 | 0.13 | 0.71 | HLA-A*11:01 |

***** Epitopes were predicted using NetMHCpan EL 4.1. Strong binders were defined as %Rank ≤ 0.5.

Table S5: Promiscuous CTL epitopes predicted to bind three HLA class I alleles.

| **Epitope (9-mer)** | **No. of HLA alleles** | **HLA restrictions** | **Best %Rank** | **Start position** |
| --- | --- | --- | --- | --- |
| TVDIRVQRL | 3 | HLA-A02:01; HLA-B07:02; HLA-B*08:01 | 0.16 | 233 |
| YRHQNRHQF | 3 | HLA-A24:02; HLA-B08:01; HLA-B*35:01 | 0.32 | 262 |
| APFDVGHRA | 3 | HLA-B07:02; HLA-B35:01; HLA-B*51:01 | 0.44 | 205 |
| AEHKQTQTL | 3 | HLA-B07:02; HLA-B08:01; HLA-B*15:01 | 0.70 | 222 |
| EHKQTQTLF | 3 | HLA-A24:02; HLA-B08:01; HLA-B*35:01 | 0.77 | 223 |

Table S6: Predicted strong-binding MHC-II (HLA-DR) 15-mer epitopes for AKOBGLPP_01618.

| **Peptide (15-mer)** | **Best %Rank** | **Strong-binding alleles (rank)** | **No. DR alleles (SB ≤ 2)** | **Source Segment** |
| --- | --- | --- | --- | --- |
| RDQYRHQNRHQFSAH | 0.04 | HLA-DRB1*13:02 (0.04); HLA-DRB1*11:01 (1.50) | 2 | 5 |
| QRDQYRHQNRHQFSA | 0.06 | HLA-DRB1*13:02 (0.06); HLA-DRB1*11:01 (1.90) | 2 | 5 |
| TGFQYALEPPRHQPE | 0.26 | HLA-DRB5*01:01 (0.26); HLA-DRB1*04:01 (1.70) | 2 | 2 |
| GDGFAPFDVGHRAAG | 0.27 | HLA-DRB5*01:01 (0.27); HLA-DRB1*13:02 (1.10) | 2 | 4 |
| IPADFAADGGIGQEI | 0.43 | HLA-DRB3*01:01 (0.43); HLA-DRB1*04:01 (0.44) | 2 | 2 |
| QGDGFAPFDVGHRAA | 0.44 | HLA-DRB5*01:01 (0.44); HLA-DRB1*13:02 (1.70) | 2 | 4 |
| HRRIRRQENENRPPS | 0.52 | HLA-DRB4*01:01 (0.52); HLA-DRB5*01:01 (0.83) | 2 | 2 |
| PADFAADGGIGQEIL | 0.54 | HLA-DRB3*01:01 (0.54); HLA-DRB1*04:01 (0.61) | 2 | 2 |
| HIPADFAADGGIGQE | 0.6 | HLA-DRB3*01:01 (0.60); HLA-DRB1*04:01 (1.10) | 2 | 2 |
| QHRRIRRQENENRPP | 0.63 | HLA-DRB4*01:01 (0.63); HLA-DRB5*01:01 (1.10) | 2 | 2 |
| GFQYALEPPRHQPEH | 0.8 | HLA-DRB5*01:01 (0.80); HLA-DRB1*04:01 (1.50) | 2 | 2 |
| DDDYSHACAERLYRT | 1.1 | HLA-DRB1*07:01 (1.10); HLA-DRB1*01:01 (1.30) | 2 | 3 |
